# Retraction: Biomarkers in Acute Traumatic Brain Injury: A Systematic Review and Meta-Analysis

**DOI:** 10.7759/cureus.r210

**Published:** 2025-12-09

**Authors:** Adarsh Kumar Singh, Shafaque Asif, Deepika Kumari Pandey, Akash Chaudhary, Vishwas Kapoor, Pawan Kumar Verma

**Affiliations:** 1 Department of Biotechnology, Centre of BioMedical Research (CBMR) Sanjay Gandhi Postgraduate Institute of Medical Sciences, Lucknow, IND; 2 Department of Molecular Medicine, Sanjay Gandhi Postgraduate Institute of Medical Sciences, Lucknow, IND; 3 Department of Neurosurgery, Sanjay Gandhi Postgraduate Institute of Medical Sciences, Lucknow, IND; 4 Department of Biostatistics and Health Informatics, Sanjay Gandhi Postgraduate Institute of Medical Sciences, Lucknow, IND

This article has been retracted by the Editors-in-Chief due to the presence of major methodological errors that invalidate the findings. The meta-analysis included a non-human (rat) study (reference 19) as if it were human clinical data. When this study is removed, only one human Tau study remains. With only a single study available, a pooled Tau AUC cannot be calculated, and the central claim that Tau is the best early biomarker is not supported. Furthermore, several inconsistencies were identified, including a discrepancy between the reported total patient sample size and the source data, numerous incorrect citations, and significant portions of the data and discussion lacking proper support.

